# Functional Neurokinin and NMDA Receptor Activity in an Animal Naturally Lacking Substance P: The Naked Mole-Rat

**DOI:** 10.1371/journal.pone.0015162

**Published:** 2010-12-21

**Authors:** Antje Brand, Ewan St. J. Smith, Gary R. Lewin, Thomas J. Park

**Affiliations:** 1 Laboratory of Integrative Neuroscience, Department of Biological Sciences, University of Illinois at Chicago, Chicago, Illinois, United States of America; 2 Department of Neuroscience, Max-Delbrück Center for Molecular Medicine, Berlin, Germany; Pontifical Catholic University of Rio Grande, Brazil

## Abstract

Naked mole-rats are extremely unusual among mammals in that their cutaneous C-fibers lack the neuropeptide Substance P (SP). In other mammals, SP plays an important role in nociception: it is released from C-fibers onto spinal neurons where it facilitates NMDA receptor activity and causes sensitization that can last for minutes, hours or days. In the present study, we tested the effects of intrathecal application of: 1) SP, 2) an SP antagonist (GR-82334), and 3) an NMDA antagonist (APV) on heat-evoked foot withdrawal. In the naked mole-rat, at a high enough concentration, application of SP caused a large, immediate, and long-lasting sensitization of foot withdrawal latency that was transiently reversed by application of either antagonist. However, neither SP nor NMDA antagonists had an effect when administered alone to naïve animals. In contrast, both antagonists induced an increase in basal withdrawal latency in mice. These results indicate that spinal neurons in naked mole-rats have functional SP and NMDA receptors, but that these receptors do not participate in heat-evoked foot withdrawal unless SP is experimentally introduced. We propose that the natural lack of SP in naked mole-rat C-fibers may have resulted during adaptation to living in a chronically high carbon dioxide, high ammonia environment that, in other mammals, would stimulate C-fibers and evoke nocifensive behavior.

## Introduction

Naked mole-rats naturally lack neuropeptides in cutaneous sensory neuron C-fibers involved in the transmission of noxious stimuli; so called nociceptors [1]. These neuropeptides include Substance P (SP) and calcitonin gene related peptide (CGRP). In other mammals, release of these neuropeptides onto spinal/trigeminal neurons is associated with increased neuronal activity and enhanced nocifensive behaviors [2]–[4]. Consistent with a lack of neuropeptides in C-fiber nociceptors, naked mole-rats display a lack of nocifensive behavior in response to certain noxious stimuli (capsaicin, acid and ammonia) and do not develop thermal hyperalgesia [5], [6], types of nociception that are associated with C-fiber activity and neuropeptide release [2], [7], [8]. Furthermore, histamine does not evoke scratching in naked mole-rats [9], the pruritogencity of which is also dependent upon capsaicin-sensitive C-fibers [10]. In contrast, naked mole-rats respond similarly to mice in acute mechanical and thermal nociceptive tests [5].

Although cutaneous C-fibers lack SP, one neuropeptide receptor is expressed in the superficial dorsal horn of the naked mole-rat spinal cord: the neurokinin 1 receptor (NK1) [5]. This suggested that spinal neurons in naked mole-rats might be capable of responding to SP in the context of nociceptive processing after experimental introduction of SP. Indeed, after intrathecal (i.t.) SP infusion naked mole-rats do display nocifensive behavior in response to capsaicin [5] and scratching in response to histamine [9]. In a second manipulation we administered a herpes virus engineered to express the preprotachykinin gene, which produces SP. Thereafter, naked mole-rats displayed capsaicin-mediated thermal hyperalgesia that was not evident before virus-driven SP expression [5]. Hence, it appears that NK1 receptors in the spinal cord of naked mole-rats participate in the processing of noxious stimuli when SP is experimentally introduced.

As part of the study described above, we presented data on the effects of i.t. application of SP on heat-evoked foot withdrawal. In mice, i.t. application of SP causes sensitization to heat in a similar fashion to the capsaicin-mediated thermal hyperlagesia described above. Post-SP administration, we found that naked mole-rats also display decreased foot withdrawal latency, e.g. sensitization [5]. In the present study we extended these findings by examining the effects of an SP receptor antagonist, administered alone or in combination with SP. Furthermore, because of evidence that SP mediates sensitization by facilitating NMDA receptor activity in spinal neurons [3], [11]–[13], we also tested the effects of an NMDA antagonist, alone and in sequential combination with SP. We find that although naked mole-rats have retained functional SP and NMDA receptors, these receptors do not play a role in heat-evoked foot withdrawal unless SP is experimentally introduced. In contrast, both these receptors play a prominent role in heat-evoked foot withdrawal in mice.

## Methods

### Ethics Statement

All animal protocols were approved by the University of Illinois at Chicago Institutional Animal Care and Use Committee (Animal Welfare Assurance Number A3460-01, Protocol Number 09–257) or the German federal authorities (State of Berlin, Approval Number Lewin 0039/99) as appropriate.

### Animals

We used 3–6 month-old, male C57BL/6 mice and 6–18 month-old naked mole-rats (*Heterocephalus glaber*). Maximum life span in naked mole-rats approaches 30 years and the mole-rats used here were thus considered to be young adults [14].

### Foot withdrawal to heat

**Figure 1:**
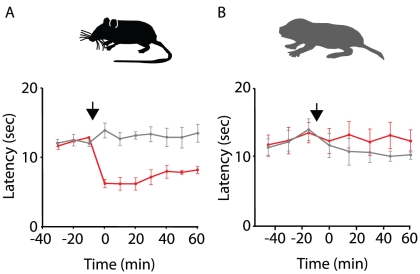
Capsaicin sensitizes heat-evoked foot withdrawal to heat in mice, but not naked mole-rats. Latencies were measured every 10 minutes in mice (A, n = 6) and naked mole-rats (B, n = 6). Saline (i.t., gray traces) or capsaicin (1 mM topical, red traces) were given after the first three time points (black arrows). Whereas saline had no effect upon withdrawal latency in either species, capsaicin induced faster foot withdrawal in mice (A), but had no effect upon naked mole-rats (B).

Animals were lightly anesthetized with Pentobarbital (35 mg/kg, i.p). Heat stimuli were generated with the focused light beam from a projection bulb calibrated to increase skin temperature to approximately 45°C over 20 seconds; such a slow heating protocol predominantly activates C-fibers [15], [16]. Stimulation was terminated after limb withdrawal or, in the absence of a withdrawal, after 20 sec. For each animal, measures of foot withdrawal latency were averaged for the medial and lateral surface of each hind foot. Testing was repeated every 10 minutes and the data from individual animals was averaged at each time point to generate a withdrawal latency curve as a function of test time for each experimental group of mice and naked mole-rats. The grey traces in Fig. 1 illustrate heat-evoked foot withdrawal latencies before and after i.t. saline administration for mice (A) and naked mole-rats (B). Note that these grey curves are used in subsequent figures to compare saline injections with drug injections. Data were collected from 6 animals in each experiment and are presented as mean ± SEM.

### Intrathecal application

SP (1 µM, 10 µM, and 100 µM; Sigma), the NK1 receptor antagonist GR-82334 (10 nM, 100 nM, and 1 µM; Tocris), and the NMDA antagonist APV (10 nM, 100 nM, and 100 µM; Tocris) were administered to animals intrathecally between vertebrae L4 and L5 (20 µl in physiological saline).

### Immunohistochemistry

Animals were anesthetized with Ketavet (Pfizer), co-administered with the muscle relaxant Rompun (Bayer), and then perfused intracardially with PBS followed by 4% PFA. The lumbar spinal cord was removed, post-fixed in 4% PFA for 30 minutes at 4°C and osmotically dehydrated overnight in 30% sucrose at 4°C. 20 µm transverse sections were subsequently processed using a cryostat CM 300 (Leica) for immunostaining. Sections were stained using an anti-NR1 primary antibody (Santa Cruz Biotechnology, SC-1467), which was detected with an anti-goat secondary antibody conjugated to Cy2. Staining was visualized using a Leica DM 5000B microscope and MetaVue software (Visitron).

## Results

### Capsaicin fails to sensitize responses to heat in naked mole-rats

Capsaicin, which is lipid soluble and hence able to penetrate the skin, is a specific agonist for the TRPV1 ion channel, expressed by many C-fibers [17]. Activation of nociceptors by capsaicin binding to TRPV1 triggers the release of glutamate and neuropeptides, including SP, onto spinal neurons. Subsequently, the area of skin that was treated with capsaicin becomes hypersensitive to heat stimuli [18], [19]. We accordingly observed that treating mice with capsaicin (1 mM) on one foot that they subsequently withdraw the treated foot from heat faster than the untreated foot: the response is sensitized (Fig. 1A). In contrast, capsaicin application did not decrease withdrawal latencies in naked mole-rats, both the capsaicin treated and untreated foot remaining at baseline levels (Fig. 1B). Repeating the experiment on naked mole-rats with a higher concentration of capsaicin (10 mM) gave the same result (data not shown).

We previously reported that NK1 receptors are expressed in the superficial dorsal horn of the spinal cord in the naked mole-rat, and that introduction of SP into the spinal cord via i.t. infusion or gene therapy rescues behavioral sensitivity to capsaicin [5]. Hence, it appears that at least part of the naked mole-rats' insensitivity to capsaicin is related to this species' natural lack of SP in its cutaneous C-fibers.

### Intrathecal application of SP sensitizes responses to heat in naked mole-rats

**Figure 2:**
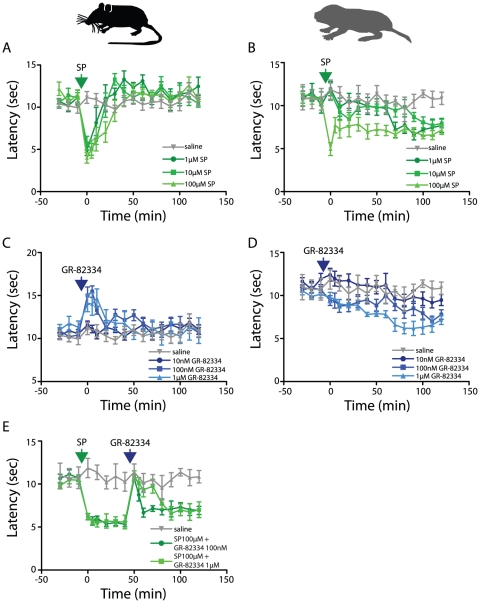
The role of SP in heat-evoked foot withdrawal. (A) SP administration (green arrow) immediately sensitizes heat-induced foot withdrawal in mice at all doses administered (green traces), whereas in naked-mole-rats (B) 1 µM and 10 µM appear to induce sensitization with a delay of ∼60 mins and only 100 µM causes immediate sensitization, an effect maintained throughout the experiment. (C) Inhibiting NK-1 receptors with GR-82334 (blue arrow) increases heat-induced foot withdrawal latency in mice at doses >100 nM (blue traces), but produces no such increase in naked mole-rats, where doses >100 nM evoke a tendency for decreased latency (D). (E) After inducing sensitization with 100 µM SP (green arrow), subsequent administration of GR-82334 (blue arrow) transiently reverses sensitization (green traces). The effect of saline (grey traces) is shown in all panels for comparison.

Intrathecal infusion of SP sensitizes withdrawal responses to heat in laboratory rats [20]–[22] and in agreement with these reports, we found that SP, at all concentrations examined (1, 10 and 100 µM), evoked a large and rapid decrease in foot withdrawal latency in mice, the duration of the effect lengthening with increasing concentration (Fig. 2A). SP also sensitized foot withdrawal responses in naked mole-rats, an effect most clearly observed at the highest dose tested (Fig. 2B). However, the response pattern for naked mole-rats differed from that of mice such that at the highest dose administered the withdrawal latency remained sensitized for the entire 120 minutes of post-SP testing. In contrast, withdrawal latencies in mice returned to baseline 30 minutes after 100 µM SP application. Another interesting difference between mice and naked mole-rats is that naked mole-rats show a minor sensitization at lower doses (Fig. 2B), but with a delay of 60–80 minutes.

### Antagonizing NK1 receptors does not inhibit heat-evoked foot withdrawal in naked mole-rats

To further explore the function of NK1 receptors in heat-evoked foot withdrawal, we investigated the effects of the SP antagonist GR-82334 at three concentrations (10 nM, 100 nM, and 1 µM). Neither species showed an effect at 10 nM GR-82334 (Fig. 2C, D), but in mice higher concentrations evoked a large and immediate increase in foot withdrawal latency (Fig. 2C). In contrast, not even the higher doses of GR-82334 increased foot withdrawal latency in naked mole-rats (Fig. 2D). In fact, at the highest concentration, naked mole-rats showed a moderate decrease in response latencies over time, reaching a maximum decrease at ∼60 minutes after application (Fig. 2D).

### SP-induced sensitization is inhibited by blocking SP NK1 receptors in naked mole-rats

To determine if GR82334 could be effective in naked mole-rats after SP administration, we sensitized heat-evoked foot withdrawal responses with 100 µM SP and 40 minutes later applied 100 nM GR-82334. We observed a large, immediate, and transient return to baseline latency levels (Fig. 2E), which shows that the SP signaling pathway can be manipulated to induce sensitization of heat-evoked foot withdrawal and that inhibiting the same pathway can abolish this sensitization. This demonstrates that the lack of inhibition by GR-82334 in Fig. 2D is not due to a lack of efficacy at naked mole-rat NK1 receptors; rather it supports the notion that under physiological conditions, the heat stimulus cannot induce SP release from C-fibers since naked mole-rats lack SP and thus there is no agonist for GR-82334 to antagonize.

### Antagonizing NMDA receptors fails to inhibit responses in naïve naked mole-rats

**Figure 3:**
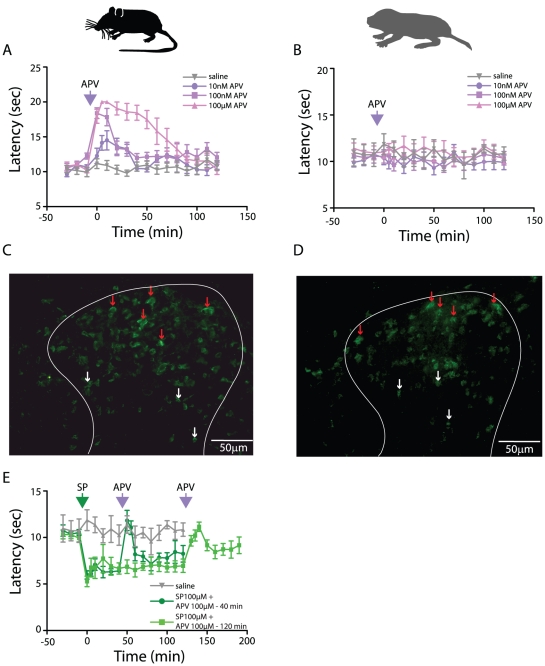
The role of NMDA-mediated neurotransmission in heat-evoked foot withdrawal. (A) The NMDA antagonist APV (pink arrow) evokes a dose-dependent increase in heat-evoked foot withdrawal latency in mice (pink traces), but has no effect on heat-evoked foot withdrawal latency in naked mole-rats. (C and D) NR1 NMDA subunit positive cells were observed in both superficial (red arrows) and deeper layers (white arrows) of the spinal cord in both mouse (C) and naked mole-rat (D). (E) After inducing sensitization with 100 µM SP (green arrow), subsequent administration of APV (100 µM, after 40 or 120 mins, pink arrows) caused a transient recovery to baseline levels of foot withdrawal latency. The effect of saline (grey traces) is shown in panels A, B and E for comparison.

It is suggested that SP initiates sensitization, at least in part, by facilitating glutamatergic, NMDA receptor-mediated activity [3], [4]. The goal of our next experiment was to determine if NMDA receptor activity, in the absence of endogenous SP, plays a role in heat-evoked foot withdrawal in naked mole-rats. To examine this, heat-evoked foot withdrawal latencies were measured before and after i.t. administration of the NMDA receptor antagonist APV. Whereas mice showed a moderate increase in foot withdrawal latency at 10 nM and a greater, more prolonged increase at higher concentrations (Fig. 3A), APV had no substantial effect at any concentration in naked mole-rats (Fig. 3B). Thus it would appear that NMDA activity plays a prominent role in heat-evoked foot withdrawal in mice, but not in naked mole-rats.

To exclude the possibility that a lack of NMDA receptors is responsible for the lack of effect of APV in naked mole-rats, we analyzed the expression of the NMDA receptor NR1 subunit in the lumbar region of the spinal cord. In both mouse and naked mole-rat we observed NR1 expression throughout the dorsal spinal cord, staining being present in both superficial and deeper laminae (Fig. 3C and D). A parsimonious explanation would therefore be that the lack of effect of APV in naked mole-rats is linked to a lack of endogenous SP-mediated sensitization of NMDA receptors.

### SP-mediated sensitization is blocked by antagonizing NMDA receptors in naked mole-rats

To assess if NMDA receptors are perhaps involved in heat-evoked foot withdrawal in naked mole-rats during SP-mediated sensitization, we first administered 100 µM SP and then, either 40 or 120 minutes later, we administered 100 µM APV. SP caused a large, immediate decrease in withdrawal latency (sensitization) and under these conditions, APV produced a large, immediate, and transient increase in foot withdrawal latency back to baseline levels (Fig. 3E).

This result indicates that the lack of effect of NMDA antagonist alone (Fig. 3B) was not due to an insufficient concentration of the antagonist. Instead, APV-mediated reversal of SP-induced sensitization suggests that, while NMDA receptor activity is not involved in baseline foot withdrawal responses, it is critical for the sensitized responses induced by SP.

## Discussion

The main findings of this study are: 1) in contrast to mice, neither endogenous SP nor NMDA receptor tone appear to modulate heat-evoked foot withdrawal in naïve naked mole-rats; 2) pre-treating naked mole-rats with i.t. SP sensitizes spinal circuits that underlie heat-evoked foot withdrawal; and 3) SP-induced sensitization in naked mole-rats can be reversed by antagonizing either NK1 or NMDA receptors.

In this study, a slow rate of heating was used to evoke foot withdrawal (∼1°C/sec), which has been demonstrated in rats to produce a foot withdrawal behavior that is mediated predominantly by C-fibers, whereas fast heating rates (>6°C/sec) evoke an Aδ-fiber-dependent foot withdrawal [15], [16]. In view of this, it is likely that differences in the properties of C-fiber nociceptors, between mice and naked mole-rats, are responsible for the differences we observed between the two species. We have previously identified several peculiarities in naked mole-rat C-fibers, compared to other mammalian species studied to date; these include: a lack of neuropeptides associated with nociception, physiological insensitivity to low pH, and an altered connectivity pattern of C-fiber nociceptors in the dorsal horn [5], [1], [7]. These traits, combined with the findings of the present study, suggest that C-fibers in naked mole-rats fail to induce sensitization of the spinal circuitry involved in thermal hyperalgesia, even though they fire action potentials to heat (and capsaicin) and can drive activity in dorsal horn neurons [5].

Intrathecal SP has been previously demonstrated to generate thermal hyperalgesia in rats [20]–[22] and we observed a similar SP-mediated sensitization of heat-evoked foot withdrawal in both mice (Fig. 2A) and naked mole-rats (Fig. 2B). This is expected because exposing dorsal horn neurons to SP would increase the level of neuronal depolarization due to the binding of SP to NK1 receptors, which leads to phospholipase C (PLC) activation and subsequent IP_3_-mediated calcium release [3]. Increased depolarization would consequently lead to the removal of voltage-dependent block of NMDA receptors [23] and PLC also, via diacyl-glycerol, activates protein kinase C [21], [24], which phosphorylates and sensitizes NMDA receptors [25], [26] in addition to increasing AMPA receptor trafficking [27]. Due to these transcription-independent phenomena, glutamate released by heat stimuli subsequent to i.t. SP administration would more readily activate NMDA and AMPA receptors leading to increased action potential firing in comparison to before SP administration and thus produce more rapid foot withdrawal. The fact that under basal conditions heat-evoked foot withdrawal latency is fairly stable (grey traces Fig. 1) suggests that activation of AMPA receptors alone is not enough to drive sensitization and indeed in rat dorsal horn neurons, inhibiting AMPA receptors with NBQX has also been observed to only decrease sensitization at high doses under non-inflammatory conditions [28].

Unlike in mice, where inhibiting NK1 receptors increases heat-evoked foot withdrawal latency (Fig. 2C), we observed that antagonizing NK1 receptors in naïve naked mole-rats had no effect on foot withdrawal latency (Fig. 2D) as would be expected due to the lack of endogenous SP in cutaneous sensory neurons [1]. However, inhibiting NK1 receptors after i.t. SP administration would prevent the spinal circuit sensitization events described above from occurring and thus after i.t. SP administration in the naked mole rat one would predict that NK1 receptor inhibition resulted in an increase in foot withdrawal latency, which is what we observed (Fig. 2E). This shows that NK1 receptors function normally in the naked mole-rat, which is expected because although SP is absent from naked mole-rat cutaneous C-fibers, both SP and CGRP have been identified in presumptive sensory fibers in the viscera [5]. The presence of SP and CGRP in some sensory afferents suggests that peptidergic neurotransmission does occur in the naked mole-rat and thus one would expect normal function of the corresponding receptors, which is what we were able to observe here via i.t. administration of SP.

The observation that blocking NMDA receptors increases heat-evoked foot withdrawal latency from basal levels in mice (Fig. 3A), suggests that some of this behavior is mediated by glutamate binding to NMDA receptors. Indeed, in rat spinal neurons inhibiting NMDA receptors has been shown to decrease heat-evoked responses [29] showing the importance of NMDA signaling in heat sensitivity in some rodents. This could be due to SP signaling, which would induce increased NMDA receptor tone, or that NMDA receptors are to some degree basally active, independent of SP-mediated sensitization. In contrast, the NMDA receptor antagonist APV had no effect on naïve naked mole-rats (Fig. 3B), even though, as in mice, naked mole-rats express NMDA receptors in the superficial dorsal horn (Fig. 3C and D). This is likely to be due to the lack of endogenous SP release, which would result in the level of NMDA activation being minimal (due to the lack of SP-mediated NMDA receptor sensitization as explained previously). However, following i.t. SP administration and sensitization of heat-evoked foot withdrawal, APV was observed to increase withdrawal latency to basal levels, thus showing that NMDA receptors are functional in naked mole-rats and play a role in processing noxious input, but that without SP-mediated sensitization, NMDA tone is probably close to zero. This suggests that heat-evoked foot withdrawal in naked mole-rats is largely mediated via AMPA receptors and the presence of AMPA-mediated excitatory post-synaptic potentials in response to a noxious stimulus in the superficial dorsal horn of naked mole-rat spinal cords is something we have previously documented [5].

We can only conjecture as to why naked mole-rats lack neuropeptides and are behaviorally insensitive to certain forms of C-fiber stimulation. Our working hypothesis is that these traits may be adaptations for living in a high carbon dioxide, high ammonia environment [30]. Naked mole-rats have a very unusual lifestyle in that they combine a fully subterranean existence with extreme sociality and a proclivity for living in colonies with many (hundreds) individuals [31], [30]. The levels of carbon dioxide (acidosis) and ammonia resulting from many animals sharing a cramped, unventilated space would be expected to stimulate trigeminal C-fibers and trigger a painful burning in the eyes and nose [32], [33]. The lack of neuropeptides from C-fibers in spinal nerves innervating the skin of the body may therefore be an epiphenomenon related to the anatomical homology of dorsal root ganglia and trigeminal ganglia [34].

In conclusion, we provide evidence for heat-evoked foot withdrawal involving both NK1 and NMDA receptors in mice, whereas neither receptor type is involved in this behavior in naïve naked mole-rats. Intrathecal SP induces sensitization of heat-evoked foot withdrawal in both species and in this sensitized state inhibition of either NK1 or NMDA receptors reverses the sensitization in naked mole-rats. We propose that the lack of endogenous SP in naked mole-rats means that heat-evoked foot withdrawal is only mediated by AMPA receptors in naïve animals, but that in the presence of SP, NMDA receptor sensitization leads to their involvement in nociceptive signaling and therefore inhibition of either NK1 or NMDA receptors reverses SP-induced sensitization.
